# Ocean acidification and desalination: climate-driven change in a Baltic Sea summer microplanktonic community

**DOI:** 10.1007/s00227-018-3321-3

**Published:** 2018-03-09

**Authors:** Angela Wulff, Maria Karlberg, Malin Olofsson, Anders Torstensson, Lasse Riemann, Franciska S. Steinhoff, Malin Mohlin, Nina Ekstrand, Melissa Chierici

**Affiliations:** 1 Department of Biological and Environmental Sciences, University of Gothenburg, Box 461, 405 30 Göteborg, Sweden; 2 Department of Marine Sciences, University of Gothenburg, Box 461, 405 30 Göteborg, Sweden; 3 Department of Biology, Marine Biological Section, University of Copenhagen, Strandpromenaden 5, 3000 Helsingør, Denmark; 4 Swedish Meteorological and Hydrological Institute, Sven Källfelts gata 15, 426 71 Västra Frölunda, Sweden; 5 Present Address: School of Oceanography, University of Washington, Box 357940, Seattle, WA 98195 USA; 6 Present Address: Institute of Marine Research, Sykehusveien 23, Tromsø, Norway

## Abstract

Helcom scenario modelling suggests that the Baltic Sea, one of the largest brackish-water bodies in the world, could expect increased precipitation (decreased salinity) and increased concentration of atmospheric CO_2_ over the next 100 years. These changes are expected to affect the microplanktonic food web, and thereby nutrient and carbon cycling, in a complex and possibly synergistic manner. In the Baltic Proper, the extensive summer blooms dominated by the filamentous cyanobacteria *Aphanizomenon* sp., *Dolichospermum* spp. and the toxic *Nodularia spumigena* contribute up to 30% of the yearly new nitrogen and carbon exported to the sediment. In a 12 days outdoor microcosm experiment, we tested the combined effects of decreased salinity (from 6 to 3) and elevated CO_2_ concentrations (380 and 960 µatm) on a natural summer microplanktonic community, focusing on diazotrophic filamentous cyanobacteria. Elevated *p*CO_2_ had no significant effects on the natural microplanktonic community except for higher biovolume of *Dolichospermum* spp. and lower biomass of heterotrophic bacteria. At the end of the experimental period, heterotrophic bacterial abundance was correlated to the biovolume of *N. spumigena.* Lower salinity significantly affected cyanobacteria together with biovolumes of dinoflagellates, diatoms, ciliates and heterotrophic bacteria, with higher biovolume of *Dolichospermum* spp. and lower biovolume of *N. spumigena*, dinoflagellates, diatoms, ciliates and heterotrophic bacteria in reduced salinity. Although the salinity effects on diatoms were apparent, they could not clearly be separated from the influence of inorganic nutrients. We found a clear diurnal cycle in photosynthetic activity and pH, but without significant treatment effects. The same diurnal pattern was also observed in situ (*p*CO_2_, pH). Thus, considering the Baltic Proper, we do not expect any dramatic effects of increased *p*CO_2_ in combination with decreased salinity on the microplanktonic food web. However, long-term effects of the experimental treatments need to be further studied, and indirect effects of the lower salinity treatments could not be ruled out. Our study adds one piece to the complicated puzzle to reveal the combined effects of increased *p*CO_2_ and reduced salinity levels on the Baltic microplanktonic community.

## Introduction

The Baltic Sea, one of the largest brackish-water bodies in the world, represents an ecosystem highly influenced by eutrophication, mediated especially by anthropogenic nutrient loading (Gustafsson et al. 2012; Kahru and Elmgren 2014). The central Baltic Sea has been proposed to be subject for a discontinuous regime shift due to climate change in combination with anthropogenic pressure (Möllmann et al. 2009). Expected effects of climate change may compromise the Baltic Sea as a recreational and economical resource and there are strong indications that an increased frequency or duration of cyanobacteria summer blooms will cause serious harm to, e.g. tourism industries (Hasselström 2008), and additional impact on ecosystem health, e.g. oxygen depletion (Kabel et al. 2012).

Over the next 100 years, the Baltic Sea is expected to undergo a temperature increase by 2–5 °C and experience increased precipitation rates, presumably leading to decreased salinity (HELCOM 2013). Concomitantly, the concentration of atmospheric CO_2_ will increase from current values of ca. 390 µatm up to > 970 µatm by year 2100 (Meehl et al. 2007; IPCC 2013). These changes will likely affect all trophic levels of the planktonic food web. Thereby, nutrient and carbon cycling will be affected in a complex and possibly synergistic manner. Due to the complexity of the systemic response, bi- and multifactorial approaches rather than single-factor experiments (Havenhand 2012; Lindh et al. 2013; Karlberg and Wulff 2013; Eichner et al. 2014a; Riebesell and Gattuso 2015) are required to decipher linkages between particular environmental changes and responses at various trophic levels of the food web.

In the Baltic Proper, the extensive summer blooms of cyanobacteria contribute up to 30% of the yearly new nitrogen and carbon exported to the sediment (HELCOM 2007), and the blooms are dominated by the filamentous taxa *Aphanizomenon* sp., *Dolichospermum* spp. (formerly *Anabaena* spp.) and the toxic *Nodularia spumigena*. Cyanobacterial nitrogen is assimilated and transferred in Baltic food webs directly through grazing, or indirectly through bioavailable nitrogen exuded from cyanobacterial cells (Ploug et al. 2010, 2011; Karlson et al. 2015). Due to group-specific differences in carbon uptake and saturation states of photosynthetic rates, increased CO_2_ concentrations will affect photosynthesis (Raven et al. 2005; Reinfelder 2011) as well as phytoplankton community composition (Bermúdez et al. 2016). In laboratory experiments using filamentous Baltic cyanobacteria, here *N. spumigena*, the effects of elevated CO_2_ levels on growth ranged from decreased growth (Eichner et al. 2014b) to increased growth rate (Wannicke et al. 2012). However, lack of effects has also been reported for *N. spumigena*, *Aphanizomenon* sp. (Karlberg and Wulff 2013) and *Dolichospermum* spp. (Brutemark et al. 2015). For heterotrophic bacteria, theoretically, increased CO_2_ levels should probably not show any direct effects (Joint et al. 2011), but direct effects with higher bacterial abundance at elevated *p*CO_2_ have nevertheless been shown (Endres et al. 2014). Increased phytoplankton biomass or productivity mediated by elevated *p*CO_2_ may stimulate growth of particle-associated bacteria (Grossart et al. 2006; Engel et al. 2013). Hence, consequences of elevated *p*CO_2_ levels for bacterioplankton are indeed difficult to predict.

Although the Baltic diazotrophic filamentous cyanobacteria seem to tolerate a wide salinity range, some differences between species have been reported. Compared to *Aphanizomenon* sp. (Lehtimäki et al. 1997; Laamanen et al. 2002), the toxic *N. spumigena* seems to tolerate a wider salinity range (5–30) with a biomass peak at salinity 10 (Lehtimäki et al. 1997) or 7 (Mazur-Marzec et al. 2005) *Anabaena* spp. (now *Dolichospermum* spp.) seems to prefer lower salinities and showed both higher growth rates and toxin concentrations at salinity 1–2 relative to salinity 5–6 (Engström-Öst et al. 2011). For heterotrophic bacteria, surface waters of the central Baltic Sea harbour members of typical freshwater bacterial groups and lack several typical marine taxa (Riemann et al. 2008; Herlemann et al. 2011). Hence, the estuarine/brackish local conditions have shaped a bacterioplankton community uniquely adapted to the local salinity regime.

The aim of this study was to test impacts of the A1FI scenario (Meehl et al. 2007) on a natural Baltic microbial community, focusing on the three dominating filamentous cyanobacteria species during the summer bloom. This scenario projects increased atmospheric CO_2_ levels (from 380 to 960 µatm), and decreased salinity (by 3 units, here from 6 to 3) for the Baltic Proper in 2100. To shed some light into the complexity of the microbial ecosystem response, we studied interactive effects of *p*CO_2_ and salinity in an outdoor experimental setup with ambient radiation and temperature conditions.

## Materials and methods

### Experimental setup

The experiment was conducted between 16 and 28 July 2010 outside of Askö Laboratory (58°49′N, 17°38′E) in the Baltic Sea. A natural community of Baltic Sea pelagic microplankton, dominated by the cyanobacterium *Aphanizomenon* sp. was collected using plankton net (mesh size 25 μm). To avoid large grazers, the collected organisms were gently filtered through a 200 μm mesh. The microbes, including organisms < 25 μm associated/attached to the phytoplankton, were inoculated in 0.2 μm filtered Baltic Sea surface water with either salinity 6 (ambient) or 3 (reduced), and divided into 4 L ultraviolet (UV) transparent Plexiglas aquaria (Mohlin and Wulff 2009). Reduced salinity was obtained by diluting natural Baltic Sea water with Milli-Q water and by compensating inorganic nutrient dilution by the addition of nutrients following N:P ratios of *f*/2 medium (Guillard 1975). The resulting nutrient concentrations on Day 0 are shown in Table 2. The aquaria were randomly placed in four basins filled with continuous flow-through seawater, exposing the microbes to natural fluctuations of temperature. Temperature in each basin was recorded with a logger (HOBO Pendant, Onset Computer Corporation, Bourne, USA). The basins were covered with green plastic mesh to reduce the irradiance, resulting in an approximate 60% reduction of the photosynthetically active radiation (PAR, 400–700 nm). This reduction was equivalent to PAR intensities at water depths of 1–2 m in the surrounding water column at the sampling site, as measured with an LI-1000 datalogger equipped with a Li-COR UWQ5201 PAR sensor (Li-COR, Lincoln, USA). A PMA2100 radiometer equipped with a 2π PMA2132 PAR sensor and a PMA2110 UV-A sensor (Solar Light, Glenside, USA) was used to record irradiances under the mesh throughout the experiment.

For each of the two salinity treatments, two partial pressures of CO_2_ (*p*CO_2_) were established by connecting each aquarium with a tube, constantly providing synthetic air (AGA Gas, Linköping, Sweden) with a *p*CO_2_ of either 380 µatm (ambient *p*CO_2_) or 960 µatm (enriched *p*CO_2_). The treatments are denoted as S6 Amb (salinity 6 and *p*CO_2_ 380 µatm), S6 High (salinity 6 and *p*CO_2_ 960 µatm) S3 Amb (salinity 3 and *p*CO_2_ 380 µatm) and S3 High (salinity 3 and *p*CO_2_ 960 µatm). The gas was dispersed to the water by ceramic air diffusers at a flow rate of ~ 15 ml min^−1^. For the *p*CO_2_ 960 µatm treatment, the effect of flow velocity (3, 9 and 15 ml min^−1^) was tested in triplicate experimental aquaria over 4 days using a culture of *N. spumigena* with a cell density corresponding to total phytoplankton cell density at Day 0. Target *p*CO_2_ was reached at 9 ml min^−1^ but 15 ml min^−1^ was chosen to compensate for an increased cell density over time. The aquaria were sealed with Plexiglas lids using silicon glue for aquaria, and small holes were maintained for gas outlet to prevent backpressure buildup. In addition, each aquarium was provided with a submerged tube connected to an external syringe, which was used to remove subsamples from the aquaria without opening the lids and, thus, disturb the *p*CO_2_ of the headspace. Quadruplicated aquaria from each of the four treatments were subsampled around 08.00 a.m. at five occasions (Days 0, 3, 5, 9 and 12) during the experiment. In addition, between Days 10 and 11, subsamples were analysed hourly for 30 h to study the diurnal cycle of pH and photosynthetic efficiency (Pulse Amplitude Modulated fluorometer, Walz Mess- und Regeltechnik, Effeltrich, Germany). After sampling, at Days 2, 5 and 9, 7 ml of *f*/2 medium without nitrate and silicate was added to every litre of the remaining sample (aiming at 0.3 µM DIP) to maintain concentrations similar to the Baltic Sea’s summer nutrient conditions. In an additional set of four aquaria manipulated with ambient levels, i.e. salinity 6 and 380 µatm CO_2_, no nutrients were added and used as a control for nutrient enrichment. To observe whether there were biological or chemical processes changing the carbonate system, one additional aquarium per treatment was set up without any microbes as a control. Because the aim of the experiment was to investigate potential combined effects of salinity and *p*CO_2_, samples from “nutrient controls” are not included in statistical analyses, but results from inorganic and particulate organic nutrient analyses, carbon chemistry and chlorophyll a (chl *a*) are shown in Tables 1, 2, 3. A field measurement of diurnal changes in pH and *p*CO_2_ was performed between Days 4 and 5; samples were taken every second hour over 24 h.

**Table 1:** Concentrations of photosynthetic pigments and biovolume of micrograzers

Day	Treatment	Chl *a*	Fucox	Myxo	Canthax	Echin	Rotifers	Ciliates
0	S6 Amb	na	na	na	na	na	2.36 (0.30)	0.02 (0.01)
	S3 Amb	na	na	na	na	na	2.46 (0.43)	0.06 (0.03)
	S6 High	na	na	na	na	na	1.52 (0.11)	0.06 (0.04)
	S3 High	na	na	na	na	na	1.82 (0.27)	0.03 (0.01)
	Control							
12	S6 Amb	3.36 (1.47)	0.13 (0.05)	0.28 (0.13)	0.16 (0.06)	0.18 (0.08)	3.00 (0.48)	0.29 (0.07)
	S3 Amb	5.68 (1.5)	0.22 (0.15)	0.88 (0.15)	0.23 (0.06)	0.43 (0.11)	2.25 (0.17)	0.25 (0.06)
	S6 High	2.55 (0.25)	0.07 (0.02)	0.44 (0.23)	0.11 (0.01)	0.13 (0.04)	2.97 (0.33)	0.56 (0.09)
	S3 High	8.71 (2.04)	0.10 (0.02)	1.03 (0.30)	0.33 (0.03)	0.64 (0.10)	2.59 (0.71)	0.24 (0.09)
	Control	1.06 (0.11)						

Pigment data are expressed as µg pigments l^−1^ for chlorophyll *a* (Chl *a*), fuxocanthin (Fucox), myxoxanthophyll (Myxo), canthaxanthin (Canthax), and echinenone (Echin). The biovolumes of two micrograzer groups (rotifers and ciliates) are expressed in mm^3^ l^−1^. The different treatments are four combinations of salinity (S6, S3) and carbon dioxide concentrations (Amb, High). Controls denote an extra treatment (S6 Amb) without nutrient enrichment. Numbers show mean values of four replicate treatments and standard error is shown between brackets

**Table 2:** Concentrations of inorganic nitrogen (DIN, NO_2_^−^ and NO_3_^−^), phosphate (DIP, PO_4_^3−^), silicic acid (Si), particulate organic carbon (POC), nitrogen (PON) and phosphorus (POP)

Day	Treatment	DIN	DIP	Si	DIN:DIP	POC	PON	POP	POC:PON	POC:PON:POP
0	S6 Amb	5.37 (0.22)	0.31 (0.02)	10.31 (0.28)	17.5 (1.19)	214 (17)	22.67 (2.18)	0.88 (0.03)	9.53 (0.63)	244:26:01
	S3 Amb	3.05 (0.37)	0.27 (0.01)	5.33 (1.01)	11.5 (0.96)	114 (31)	12.28 (3.23)	0.86 (0.02)	9.16 (0.18)	134:14:01
	S6 High	5.34 (0.06)	0.46 (0.03)	10.50 (0.54)	11.3 (0.60)	287 (59)	31.42 (5.58)	0.91 (0.02)	9.02 (0.47)	317:35:01
	S3 High	3.06 (0.18)	0.26 (0.01)	5.84 (0.24)	12.1 (0.58)	112 (14)	12.41 (1.49)	0.86 (0.02)	9.02 (0.38)	132:15:01
	Control	5.43 (0.51)	0.29 (0.02)	11.52 (0.98)	19.5 (1.34)	176 (29)	21.03 (3.54)	0.88 (0.01)	8.39 (0.08)	199:24:01
12	S6 Amb	0.20 (0.04)	0.08 (0.03)	0.61 (0.09)	2.75 (1.80)	106 (22)	11.64 (2.59)	1.45 (0.07)	9.20 (0.20)	74:08:01
	S3 Amb	0.21 (0.04)	0.11 (0.02)	0.63 (0.17)	1.67 (0.52)	46 (13)	4.66 (1.34)	1.24 (0.08)	9.53 (0.23)	38:04:01
	S6 High	0.18 (0.03)	0.23 (0.04)	0.77 (0.29)	0.75 (0.16)	151 (19)	15.99 (1.60)	1.46 (0.16)	9.39 (0.23)	108:11:01
	S3 High	0.39 (0.15)	0.09 (0.02)	0.72 (0.16)	2.93 (1.02)	129 (68)	9.90 (2.71)	1.63 (0.20)	8.34 (0.07)	93:07:01
	Control	0.20 (0.02)	0.10 (0.03)	1.75 (0.77)	1.53 (0.34)	75 (23)	7.74 (2.33)	0.84 (0.05)	9.79 (0.29)	92:09:01

All quantified data are expressed in µM. The different treatments are four combinations of salinity (S6, S3) and carbon dioxide concentrations (Amb, High). Controls denote an extra treatment (S6 Amb) without nutrient enrichment. Numbers show mean values of four replicate treatments and standard error is shown between brackets

**Table 3:** Experimental conditions and manipulations, including salinity, experimental temperature, total alkalinity (*A*_T_) and pH total scale (pH_T_)

Day	Treatment	Salinity	Temperature (°C)	*A*_T_ (µmol kg^−1^)	pH_T_
0	S6 Amb	6.0	15.3	1523 (1.6)	7.59 (0.03)
	S3 Amb	3.3	15.3	824 (5.6)	7.51 (0.03)
	S6 High	6.1	15.3	1523 (3.2)	7.59 (0.04)
	S3 High	3.2	15.3	815 (1.1)	7.44 (0.06)
	Control	6.0	15.3	1510 (0.4)	7.67 (0.01)
12	S6 Amb	6.0	15.3	1592 (3.6)	7.92 (0.02)
	S3 Amb	3.2	15.3	962 (3.8)	7.81 (0.02)
	S6 High	6.0	15.3	1629 (3.6)	7.66 (0.05)
	S3 High	3.2	15.3	1020 (4.2)	7.65 (0.01)
	Control	6.0	15.3	1582 (1.9)	7.87 (0.02)

pH_T_ and *A*_T_ are measured and presented at 25 °C. Data from Day 0 were obtained before bubbling started. The different treatments are four combinations of salinity (S6, S3) and carbon dioxide concentrations (Amb, High). Controls denote an extra treatment (S6 Amb) without nutrient enrichment. Numbers show mean values of four replicate treatments and standard error is shown between brackets

### Phytoplankton species composition and growth

At Days 0, 3, 5, 9 and 12, 50 ml from each aquarium was preserved with alkaline Lugol’s solution, stored in the dark and analysed within 3 months using the Utermöhl method according to HELCOM (2008). Half of the bottom surface of the chamber was viewed in 10× magnification (Axiovert 40CFL, Micrometer Ocular 44 42 32 E-Pl 10×/20, Zeiss, Oberkochen, Germany) and all organisms larger than 30 µm was counted and grouped, either to species level or order. The length and width of filamentous species were also measured and the biovolume (mm^3^ l^−1^) was calculated. In both 20× and 40× magnification, a diagonal of the chamber bottom was analysed and organisms including micrograzers (e.g. ciliates) no smaller than 8 µm were counted.

The growth rate (mm^3^ day^−1^) for each cyanobacteria species was calculated separately for Days 0 to 3 and Days 9 to 12. The specific growth rate (*µ* day^−1^) was calculated according to (ln *D*B − ln *D*A)/(*t*B – *t*A), where *D*A is the biovolume at the first day and *D*B the biovolume at the end of the period, *t*A is day A and *t*B is day B. For Day 12, species diversity was calculated by Shannon’s index.

### Photosynthetic pigments and nodularin

At Day 12, 100 ml from each aquarium was filtered onto 25 mm GF/F filters (Whatman, GE Healthcare, Chicago, USA) and the filters were flash frozen in liquid nitrogen. Filters were later extracted in 100% MeOH, ultrasonicated, and both the extraction and HPLC analysis followed Wright and Jeffrey (1997), described in detail in Mohlin and Wulff (2009). Pigments are expressed as concentrations (ng cell^−1^). For aphanizophyll and 4-keto-myxoxanthophyll, the response factor for myxoxanthophyll was used.

The cyanotoxin nodularin, produced by *N. spumigena*, was analysed using HPLC according to Pattanaik et al. (2010). At Days 0 and 12, 200 ml from each aquarium was filtered onto 25 mm GF/F filters (Whatman, GE Healthcare, Chicago, USA). Only intracellular nodularin was analysed. Due to concentrations at or below the detection limit, no results are presented.

### Photosynthetic activity

Photosynthetic activity was estimated by variable chlorophyll fluorescence measurements in photosystem II (PSII) with a WATER-PAM chlorophyll fluorometer calibrated for cyanobacterial application (Walz Mess- und Regeltechnik, Effeltrich, Germany). Minimum fluorescence (*F*_0_′) was determined by applying a low level of light and the maximum fluorescence (*F*_m_′) by exposing the sample to a short saturation pulse of measuring light (> 4000 μmol photons m^−2^ s^−1^ for 0.6 s). Variable fluorescence (*F*_v_ = *F*_m_′ − *F*_0_′) and effective quantum yield (Δ*F*/*F*_m_′) were determined for all samples.

### Bacterial abundance and production

Duplicate samples from each aquarium (1.5 ml) were fixed on Days 0, 3, 5, 9 and 12 with EM grade glutaraldehyde (Sigma-Aldrich, St. Louis, USA, 1% final concentration) and stored at − 80 °C. Bacterial abundance was determined by flow cytometry (FACSCanto II, BD Biosciences, San Jose, USA) after staining with SYBR Green I (Molecular Probes, Thermo Fisher Scientific, Waltham USA, Marie et al. 1997) using 1.0 µm green fluorescent polymer microspheres (Duke Scientific Corporation, Thermo Fisher Scientific, Waltham USA) as internal standard in each sample. Fluorescent beads (True counts, Becton–Dickinson, Franklin Lakes, USA) were used to calibrate the flow rate.

Bacterial productivity was measured by [^3^H]-thymidine incorporation (Fuhrman and Azam 1982) as modified for microcentrifugation by Smith and Azam (1992). From each aquarium, duplicate 1.7 ml aliquots were incubated in darkness with [^3^H]-thymidine (20 nM final concentration, GE Healthcare, Chicago, USA) in sterile 2.0 ml capacity polypropylene tubes for ca. 1 h at in situ temperature. Samples with 5% trichloracetic acid added prior to the addition of isotope served as blanks. Thymidine incorporation was converted to carbon production using 1.4 × 10^18^ cells mole^−1^ thymidine incorporated (average calculated from published Baltic Sea data, SE = 0.1 × 10^18^ cells mole^−1^ thymidine, *n *= 73, HELCOM guidelines, Helsinki Commission) and a carbon content per cell of 20 fg (Lee and Fuhrman 1987). The appropriate use of 20 nM ^3^H-thymidine was confirmed by saturation curves.

### Stoichiometry and analyses of dissolved inorganic nutrients

For analyses of particulate organic carbon (POC), nitrogen (PON) and phosphorus (POP), at Days 0 and 12, 100 ml from each aquarium was filtered onto pre-combusted (400 °C for 4 h) 25 mm GF/C filters (Whatman, GE Healthcare, Chicago, USA). The filters for POP analyses were washed with 0.1 M HCl and rinsed with Milli-Q prior to filtration. All filters were then frozen at − 20 °C and freeze-dried for 36 h (Heto Power Dry PL3000, Thermo Fisher Scientific, Waltham, USA). Filters for POC/PON analysis were ground (MM301, Retsch, Haan, Germany) and a subsample was carefully weighed and analysed in an elemental analyser (EA 1108 CHNS-O, Fisons Instruments, Thermo Fisher Scientific, Waltham USA) applying 2,5-bis-[5-ert.-butyl-bensoaxzol-2-yl]-thiophen as a standard. POP-filters were analysed at Tvärminne Zoological Station, Finland, according to Solorzano and Sharp (1980).

Samples for determination of inorganic nitrogen (DIN, NO_2_^−^ and NO_3_^−^), phosphate (DIP, PO_4_^3−^) and silicic acid (Si) concentrations (µM) were filtered through 0.45 µm pore-size polycarbonate filters, frozen in – 80 °C until analysed using colorimetric determination performed on an autoanalyser (Grasshoff et al. 1999) at the accredited laboratory of the Swedish Meteorological and Hydrological Institute, Gothenburg (Sweden).

### Determination of the carbon dioxide system

Samples for pH and total alkalinity (*A*_T_) were analysed following established protocols for seawater carbonate system determination (e.g. Dickson et al. 2007). *A*_T_ was determined by potentiometric titration (Metrohm 800 Dosino and Aquatrode with Pt1000, Metrohm, Herisau, Switzerland) in an open cell with 0.05 M hydrochloric acid (Mattsdotter-Björk et al. 2014). The precision of the *A*_T_ measurements was obtained by triplicate analysis of one sample and was estimated to ca. ± 3 µmol kg^−1^. The accuracy of *A*_T_ was ± 5 µmol kg^−1^ throughout the entire experiment and controlled using Certified Reference Material (CRM, batch 79) supplied by A. Dickson (San Diego, USA). pH was determined spectrophotometrically (diode-array spectrophotometer, HP8452, Hewlett-Packard, Palo Alto, USA) on the total scale (pH_T_) using a 2 mM solution (salinity 6) of the sulphonephtalein dye, m-cresol purple, as an indicator (Clayton and Byrne 1993). Prior to analysis, the samples were thermostated to ~ 25 °C and filtered through a 0.45 µm pore-size polycarbonate filter, to remove particles that could disturb the measurement. Samples were measured in a 1-cm cell, where the temperature was measured using a thermistor with a precision of 0.1 °C. The analytical precision was estimated to ± 0.004 pH_T_ units, which was determined by a series of ten analyses of one sample. The pH_T_ of the indicator solution was measured daily in a 0.2 mm quartz cell. The perturbation of seawater pH_T_ caused by the addition of the indicator solution was calculated and corrected for using the method described in Chierici et al. (1999). *A*_T_, pH_T_, salinity, temperature, DIP and Si concentration were used in a chemical speciation model (CO2SYS, Pierrot et al. 2006) to calculate *p*CO_2_, total inorganic carbon (*C*_T_), and pH_T_ at in situ temperature. We used the CO_2_-system dissociation constants by Mehrbach et al. (1973) as refit by Dickson and Millero (1987).

### Statistical analyses

Data were analysed with two-way ANOVA using SPSS software (PASW Statistics ver. 18, SPSS Inc., Chicago, USA) for each sampling day. Homogeneity was tested by Cochran’s test and, where needed, data were transformed according to Underwood (1997). Non-metric multidimensional scaling (nMDS) of microalgal community structure was performed on square-root-transformed relative species biovolume data from Day 12, using Bray–Curtis dissimilarity in the vegan package in R (R Core Team 2016; Oksanen et al. 2017). PERMANOVA was used for detecting differences between experimental treatments in the dissimilarity matrix, using 1000 permutations in the vegan package in R. Significant differences were set as *p* < 0.05. Correlations between concentration of heterotrophic bacteria and different species of microplankton including micrograzers (i.e. ciliates) were performed with Pearson correlation, using SPSS software as above.

## Results

In our study, salinity seemed more important than *p*CO_2_; however, possible concomitant effects of diluted nutrient concentrations will be discussed. For *p*CO_2_, only effects on biovolumes of *Dolichospermum* spp. and biomass of heterotrophic bacteria were observed. No interaction effects of salinity and *p*CO_2_ were found. The biovolume of the toxic *Nodularia spumigena* was negatively affected by salinity 3, and during the ca 2 weeks experiment the initially dominating *Aphanizomenon* sp. was replaced by *Dolichospermum* spp. The A1FI scenario (salinity 3 and *p*CO_2_ 960 µatm) resulted in increased biomass of *Dolichospermum* spp. Although time was not considered a treatment factor for the experimental design, still some differences irrespective of treatment were apparent in the different variables measured (“Successional changes irrespective of treatment", see below).

### Treatment effects

#### Biomass and community composition

**Fig. 1:**
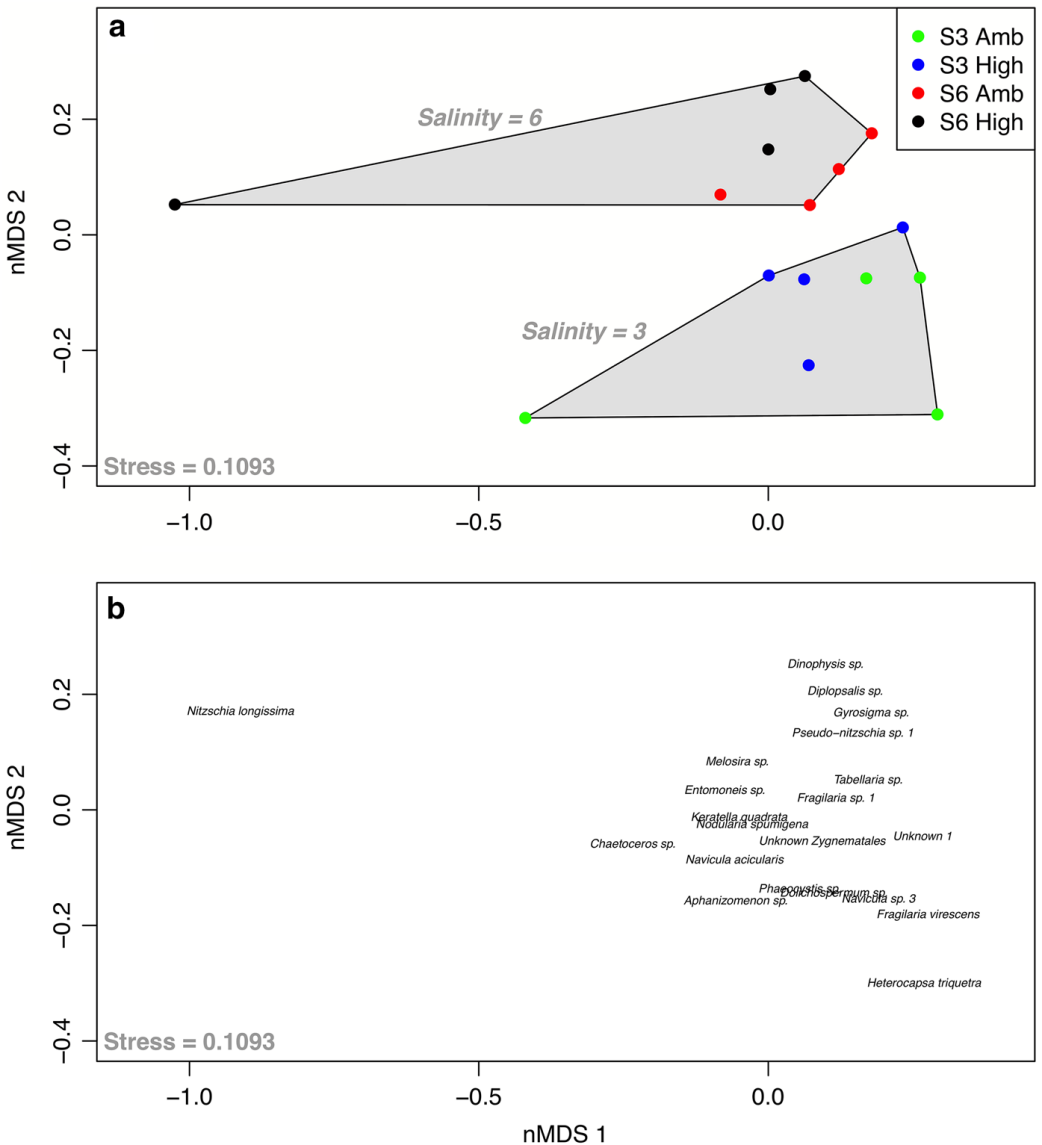
Non-metric multidimensional scaling of relative species abundance on Day 12, displayed with sample (**a**) and species (**b**) scores. The dissimilarity matrix was calculated with Bray–Curtis dissimilarity. PERMANOVA revealed that salinity treatment had a significant effect on the species composition (pseudo-*F*_(1,12)_ = 14.2, *p* < 0.001), which is illustrated with the grey ellipses in (**a**)

**Fig. 2:**
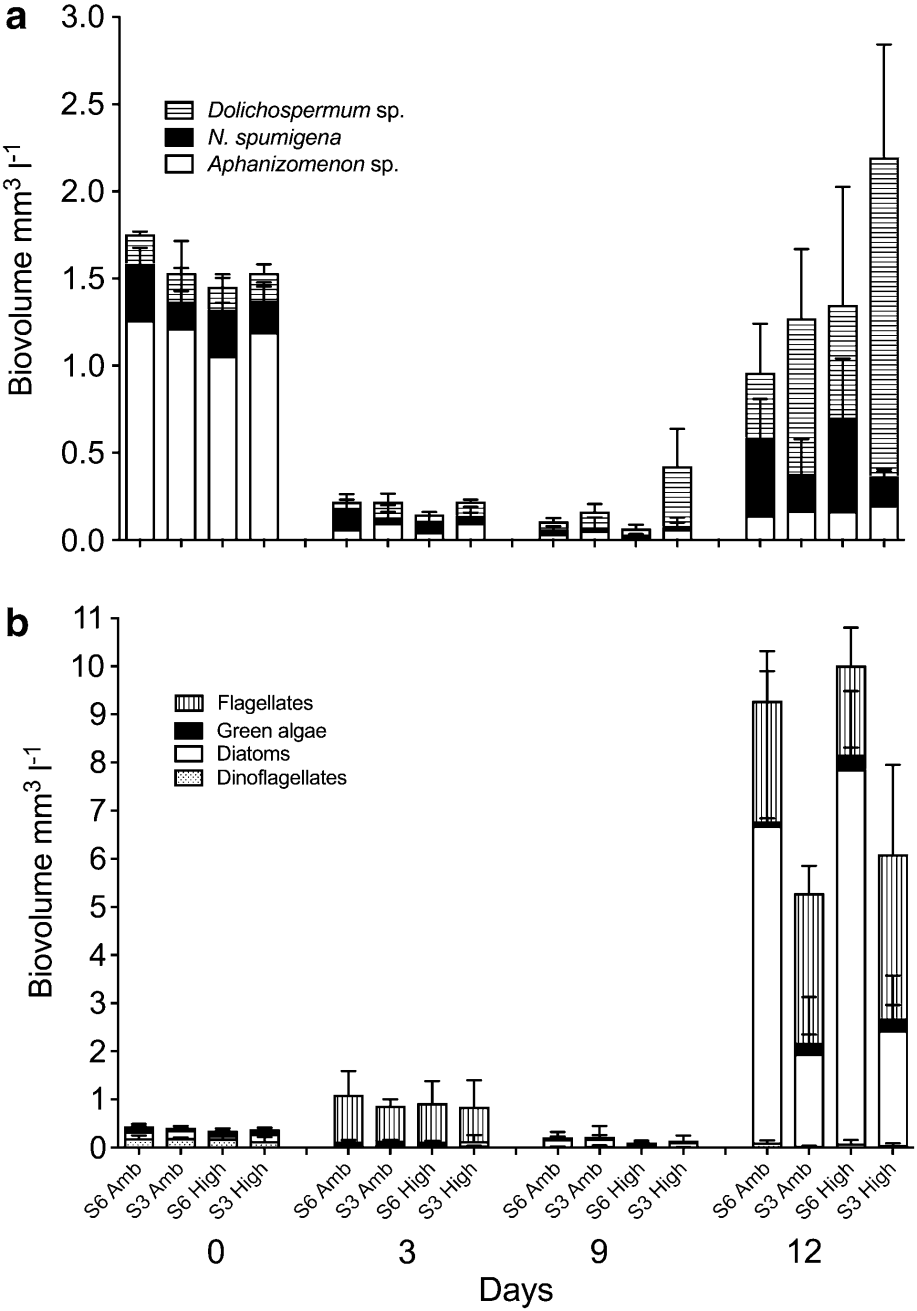
Biovolumes of diazotrophic cyanobacteria (**a**) and (**b**) biovolumes of flagellates, green algae, diatoms and dinoflagellates. The different treatments are four combinations of salinity (S6, S3) and carbon dioxide concentrations (Amb, High). Vertical lines show standard deviation (*n* = 4)

No interaction effects were found, but effects of salinity and *p*CO_2_, respectively, were observed. On Day 12, total phytoplankton biomass, approximated by chl *a*, was significantly higher in salinity 3 compared to salinity 6 (*F*_(1,12)_ = 6.63, *p* = 0.024, two-way ANOVA, Table 1). The microalgal community structure, defined as the relative species biovolume, varied significantly between the salinity treatments Day 12 (pseudo-*F*_(1,12)_ = 14.2, *p* < 0.001, PERMANOVA, Fig. 1). These differences were further investigated for specific taxa; *Dolichospermum* spp. showed significantly higher biovolumes in treatments with salinity 3 compared to present day salinity of 6 (Fig. 2). The effect of lower salinity persisted throughout the experiment (Day 12; *F*_(1,11)_ = 10.40, *p* = 0.007, two-way ANOVA). In addition, elevated *p*CO_2_ stimulated the total biovolume of *Dolichospermum* spp. (Day 12; *F*_(1,11)_ = 5.26, *p* = 0.04, two-way ANOVA), with the highest values found for the treatment with salinity 3 and elevated *p*CO_2_ (Fig. 2). For the toxic *N. spumigena*, lower biovolumes were found in salinity 3 compared to salinity 6 (Day 12; *F*_(1,11)_ = 7.21, *p* = 0.020, two-way ANOVA, Fig. 2). The biovolumes of dinoflagellates and diatoms were negatively affected by reduced salinity and showed higher biovolumes in salinity 6 by Day 12 (*F*_(1,12)_ = 8.86, *p* = 0.012 (dinoflagellates); *F*_(1,12)_ = 13.11, *p* = 0.004 (pennate diatoms); *F*_(1,12)_ = 33.04, *p* < 0.001 (centric diatoms); two-way ANOVA, Fig. 2). No significant treatment effects were observed for specific growth rate (*µ* day^−1^) of cyanobacteria except for *Dolichospermum* spp., with initially (Days 0 to 3) lower growth rate in salinity 6 compared to salinity 3 (*F*_(1,11)_ = 11.61, *p* = 0.006, two-way ANOVA). The highest growth rate of 1.2 day^−1^ was observed for *N. spumigena* between Days 9 and 12. Shannon’s index showed that the highest biodiversity (1.40) was found at salinity 6 (Day 12; *F*_(1,12)_ = 17.34, *p* = 0.001, two-way ANOVA).

The concentration of carotenoids confirmed the results from phytoplankton biovolumes with an overall dominance of pigments specific for cyanobacteria; myxoxanthophyll, canthaxanthin and echinenone. For these pigments, higher concentrations were found in salinity 3 compared to salinity 6 (Table 1). Fucoxanthin, here a proxy for diatoms, showed no treatment effects.

**Fig. 3:**
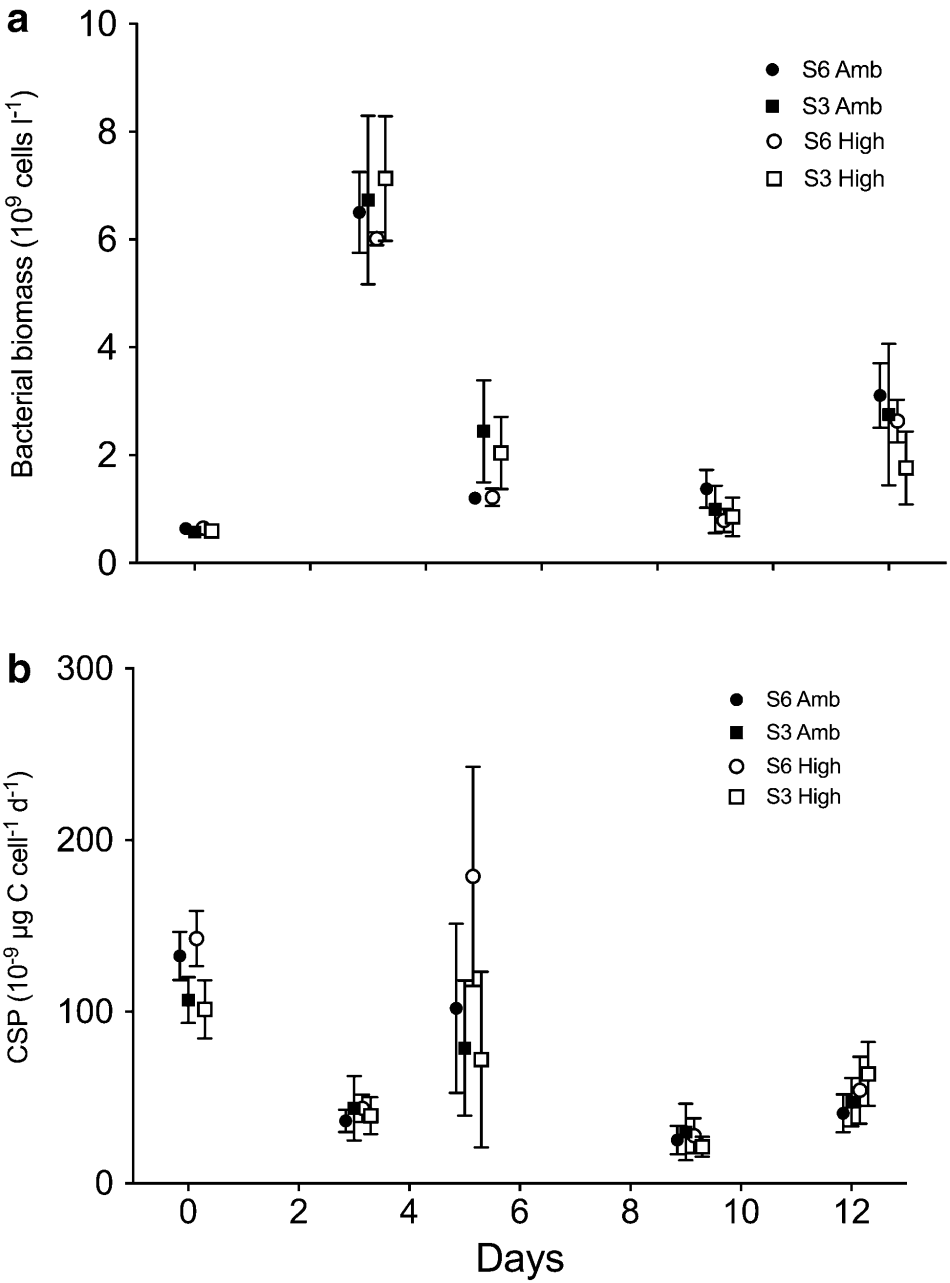
Bacterial cell numbers (**a**) and (**b**) cell-specific bacterial productivity (CSP, estimated by thymidine uptake rates). The different treatments are four combinations of salinity (S6, S3) and carbon dioxide concentrations (Amb, High). Vertical lines show standard deviation (*n* = 4). NB: Data points are positively nudged on the *x* axis to properly display the error bars

Total biovolumes of micrograzers were not affected by the treatments, but for ciliates higher biovolumes were found in salinity of 6 compared to salinity 3 (Day 12, *F*_(1,12)_) = 5.24, *p* = 0.041, two-way ANOVA, Table 1). By Day 12, the biomass of heterotrophic bacteria was significantly higher in present day conditions of salinity 6 and *p*CO_2_ 380 µatm, respectively (*F*_(1,28)_ = 5.20, *p* = 0.030; *F*_(1,28)_ = 7.39, *p* = 0.011, two-way ANOVA, Fig. 3). As a consequence of dilution to reach target salinity of 3, significant treatment effects were observed already at Day 0, with higher concentrations in salinity 6 (*F*_(1,28)_ = 64.29, *p* < 0.000, two-way ANOVA); however, the difference was only 10%. Neither bacterial productivity nor cell-specific productivity (Fig. 3) showed any treatment effects by Day 12, but initially both were higher at the present day salinity of 6 (*F*_(1,28)_ = 45.32, *p* = 0.000 and *F*_(1,28)_ = 19.28, *p* = 0.001, respectively, two-way ANOVA).

#### Stoichiometry and dissolved inorganic nutrients

No significant treatment effects were observed for either concentrations or elemental ratios (POC, PON and POP, Table 2). Treatment effects were observed for DIN, DIP and Si concentrations (Table 2). Due to dilution, dissolved inorganic nutrient concentrations were higher in aquaria with salinity 6 at Day 0 (Table 2). At Day 0 (before adding CO_2_), effects of both salinity and *p*CO_2_ were observed in the DIN:DIP ratio with a significantly higher ratio at salinity 6 in *p*CO_2_ 380 µatm (Table 2). At salinity 6, Si decreased at a higher daily rate between Days 5 and 9 compared to Days 3–5 (*F*_(1,11)_ = 26.54, *p* = 0.0009, two-way ANOVA, not shown). The Si decline was concomitant with increasing diatom biovolumes (Fig. 2). Furthermore, at Day 0, significant treatment effects were observed in the elemental ratio DIN:DIP:Si; at salinity 6 the *p*CO_2_ 380 µatm treatment showed a higher ratio than the *p*CO_2_ 960 µatm treatment, and at *p*CO_2_ 960 µatm, a higher ratio was found in salinity 3 compared with salinity 6. No statistically significant treatment effects remained at Day 12.

#### Carbon dioxide system

Initially (Day 0, before adding CO_2_), average pH_T_ in salinity 6 was 7.59 (SE 0.01) and in salinity 3, 7.47 (SE 0.03) (no significant treatment effects). From Days 3 to 12, the high *p*CO_2_ treatment had significantly higher *p*CO_2_ compared to the 380 µatm treatment, but differed from target *p*CO_2_. At Day 12, *p*CO_2_ for the 380 µatm treatment at salinity 6 was 403 µatm (SE 18) and for salinity 3, 342 µatm (SE 16). For the high *p*CO_2_ treatment, mean *p*CO_2_ was 833 µatm (SE 108) at salinity 6, and 579 µatm (SE 39) at salinity 3. The continuous supply of CO_2_ complicates interpretation of changes in pH in relation to treatments. Initial *A*_T_ was on average 1523 and 820 µmol kg^−1^ at salinities of 6 and 3, respectively (Table 3). The difference was due to the initial dilution performed to reach target salinity. At Day 12, *A*_T_ in the 380 µatm treatment was 1592 µmol kg^−1^ (SE 4) for salinity 6, and 962 µmol kg^−1^ (SE 4) for salinity 3 (Table 3). For the high *p*CO_2_ treatment, corresponding values were 1629 µmol kg^−1^ (SE 4) and 1020 µmol kg^−1^ (SE 4), for salinity 6 and 3, respectively (Table 3). The *A*_T_ increase was generally higher in the high CO_2_ treatments, regardless of salinity.

### Successional changes irrespective of treatment

#### Biomass and community composition

**Fig. 4:**
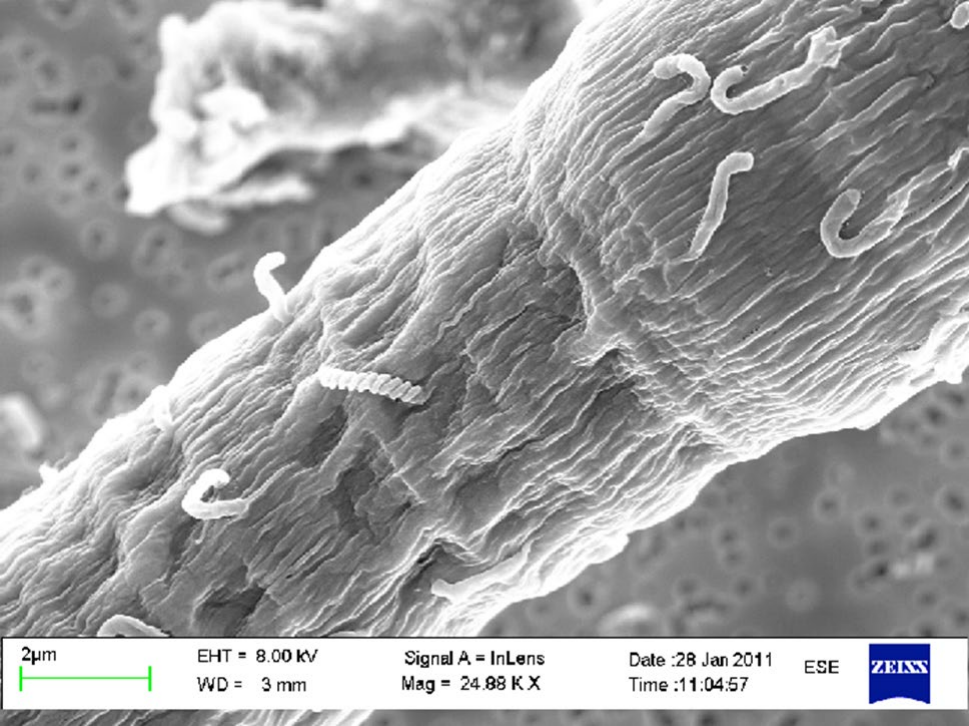
The filamentous cyanobacteria *Nodularia spumigena* with associated heterotrophic bacteria. Effects of climate change on the autotrophic community may have indirect effect on closely associated heterotrophic bacteria and their biogeochemical interactions

Changes with time were found in the structure of the microbial community, indicating a successional pattern during the experiment (not statistically tested). For phytoplankton biomass, proxied by total cell biovolumes and chl *a* concentrations, a general decline from initial values was followed by an increase until termination of the experiment by Day 12 (Table 1, Fig. 2). However, total biovolume of dinoflagellates decreased from Days 0 to 12 (Fig. 2). The most striking result was the shift in cyanobacteria composition where *Aphanizomenon* sp. decreased from the initial 87 to 15% and *Dolichospermum* spp. increased from 11 to 82% of the total filamentous cyanobacterial biovolumes. The successional pattern in phytoplankton biovolumes was mirrored by the concentration of heterotrophic bacteria; however, when phytoplankton biovolumes decreased the concentration of heterotrophic bacteria increased (Figs. 2, 3). This trend was consistent until Day 9. Between Days 9 and 12 the biomass of both phytoplankton and bacteria increased. Bacterial productivity followed the bacterial biomass with the exception that cell-specific productivity differed from bacterial biomass and declined from Days 0 to 3 (Fig. 3). At Day 12, a significant positive correlation (Pearson, *r*^2^(15) = 0.647, *p* = 0.009) between the concentration of heterotrophic bacteria and *N. spumigena* was found. An example of epiphytic bacteria associated with *N. spumigena* is shown in Fig. 4. There was no significant correlation between heterotrophic bacterial concentration and concentration of any other organisms, such as other cyanobacteria, dinoflagellates, diatoms or ciliates.

#### Stoichiometry and dissolved inorganic nutrients

POC and PON concentrations generally decreased over time, while POP concentrations increased (Table 2). For all treatments, POC:POP and PON:POP had generally lower relative Redfield ratios of 106 and 16, respectively (Day 12). In contrast, POC:PON ratios were higher than the Redfield ratio of 6.6 for all treatments throughout the experiment (Table 2). The inorganic nutrient concentrations DIN, DIP and Si decreased between Days 0 and 3 (all treatments, not shown). DIN decreased drastically between Days 0 and 3, and remained low (< 0.4 µM) throughout the remaining part of the experiment. Despite the addition of DIN, values were similar to the control treatment where no inorganic nutrients were added. The DIN:DIP ratio decreased in all treatments, including the control treatment, from Day 0 to Day 12, again showing nitrogen limitation (ratio < 3, Redfield = 16). Si decreased continuously throughout the experiment and at a faster rate towards the end of the experiment (from 0.5–1.1 µM Day 9, to 0.6–0.8 µM, Day 12), in concert with the increasing diatom biovolumes (Fig. 2). Limitation of Si was confirmed by the increased DIN:DIP:Si ratios in all treatments from Day 0 to Day 12; however, all treatments including the control treatment showed Si limitation already on Day 0 (ratio above 1.1; Brzezinski 1985).

#### Carbon dioxide system

The pH increased in all treatments during the experiment (Table 3), indicating CO_2_ uptake (net primary production) further supported by the decrease in *p*CO_2_ for all treatments (despite the continuous supply of CO_2_ to the aquaria). *A*_T_ increased in all treatments over time (Table 3). *A*_T_ is not affected by changes in *p*CO_2_ and observed changes are likely caused by net assimilation of NO_3_^−^ and H^+^ (e.g. protein synthesis during photosynthesis).

### Diurnal variations

#### Photosynthetic activity and pH in experimental aquaria

**Fig. 5:**
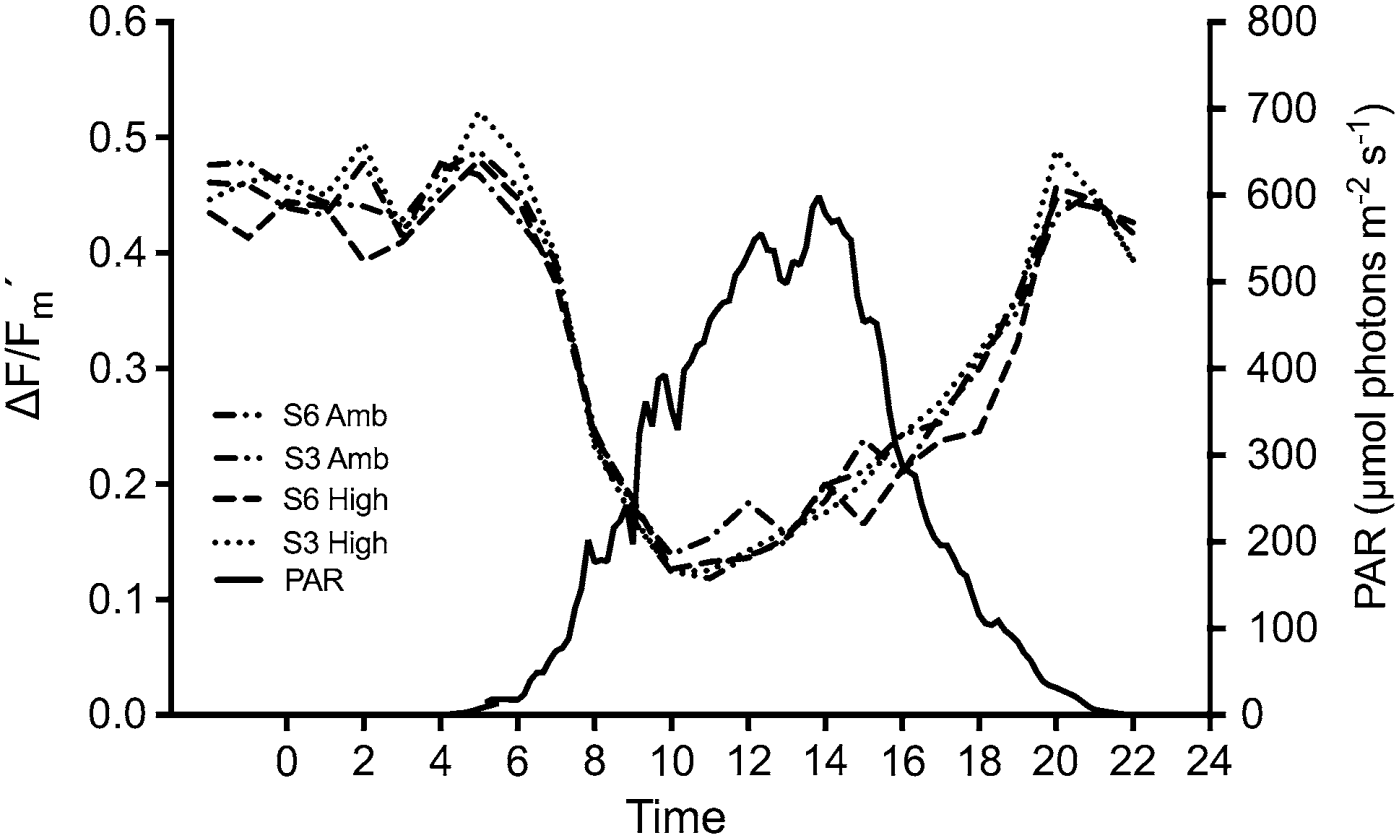
Hourly measurements of effective quantum yield (Δ*F*/*F*_m_′) during 30 h under ambient PAR (400–700 nm) conditions. Active down-regulation of photosynthesis is observed during midday trough Δ*F*/*F*_m_′ depression in all treatments. The different treatments are four combinations of salinity (S6, S3) and carbon dioxide concentrations (Amb, High)

**Fig. 6:**
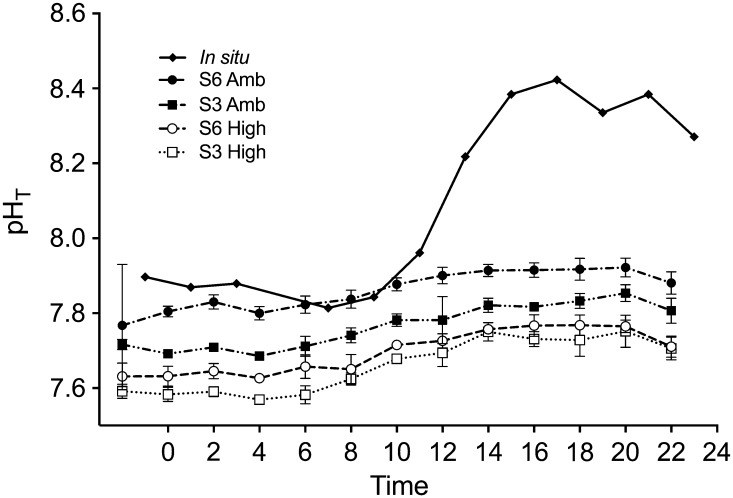
Variation in pH_T_ over 24 h. The continuous bubbling of CO_2_-enriched synthetic air provides a fluctuating pH during the diurnal cycle of primary production. The different treatments are four combinations of salinity (S6, S3) and carbon dioxide concentrations in µatm, (Amb, High) plus in situ sea surface values. Vertical lines show standard deviation (*n* = 4)

During the hourly measurements over 30 h, no significant treatment effects were found in Δ*F*/*F*_m_′. However, all treatments showed dynamic response to radiation saturation where Δ*F*/*F*_m_′ was depressed from early morning to around 17:00 (solar time). From late afternoon, the radiation stress diminished and Δ*F*/*F*_m_′ returned to original values (Fig. 5). Also, a clear diurnal pH cycle was observed, with lowest values during night/early morning (7.57) and highest values in the evening (7.92) (Fig. 6).

#### Diurnal in situ variability of pH and pCO_2_

Samples were taken every second hour for a 24-h period directly in situ to observe the natural variability outside the aquaria. We found a clear diurnal cycle related to CO_2_ uptake during photosynthesis, and pH varied from 7.79 early in the morning to 8.42 in the late afternoon (Fig. 6). The opposite pattern was observed for *p*CO_2_: the lowest value 118 µatm in late afternoon and the highest, 570 µatm in night/early morning. Mean *A*_T_ in situ was 1498 µmol kg^−1^ (SE 4).

#### Radiation and temperature

**Fig. 7:**
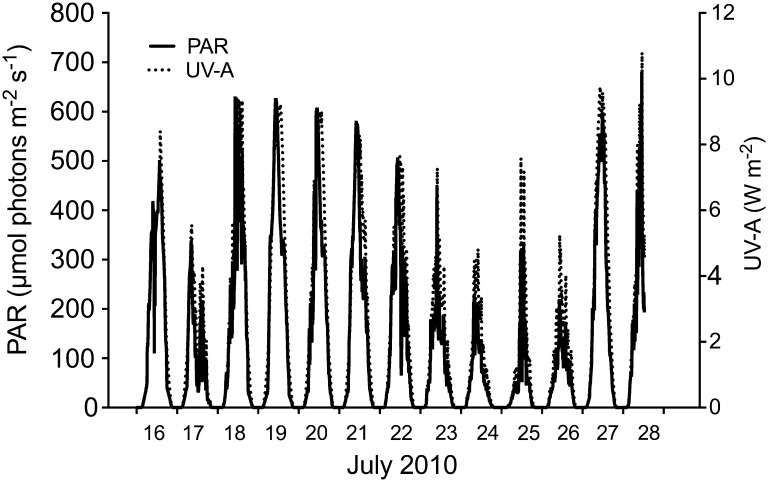
Daily variations of photosynthetic active radiation (PAR, 400–700 nm) and ultraviolet-A radiation (UV-A, 320–400 nm) during the experimental period. The aquaria and light sensors were placed under a mesh to simulate light intensities experienced in the surface water of the Baltic Sea (see “Experimental setup”)

The intensities of PAR and UV-A during the experimental period are shown in Fig. 7. Initially, sunny conditions were followed by cloudy days from Days 7 to 12. A sunny day, PAR in the water outside Askö Laboratory measured 450 µmol photons m^−2^ s^−1^ at 1 m depth, while cloudy days showed typical intensities of 180 µmol photons m^−2^ s^−1^. Experimental temperatures followed fluctuations in ambient water surface temperatures.

## Discussion

Our aim was to test the combined impact of decreased salinity (from 6 to 3) and elevated *p*CO_2_ (from 380 µatm to 960 µatm), following the A1FI scenario (Meehl et al. 2007) on a natural Baltic Sea microbial community, focusing on filamentous cyanobacteria during the summer bloom. No interaction effects of salinity and *p*CO_2_ were found. As a driver of the microbial community structure and biomass, salinity seemed more important than *p*CO_2_; however, possible concomitant effects of diluted nutrient concentrations will be discussed. Long-term effects of the experimental treatments need to be further studied, and indirect effects of the lower salinity treatments could not be ruled out. The biomass of the toxic *N. spumigena* was negatively affected by the reduced salinity, and during the experiment the initially dominating *Aphanizomenon* sp. was replaced by *Dolichospermum* spp. The shift between *Aphanizomenon* sp. and *Dolichospermum* spp. was also observed in situ. Heterotrophic bacteria seemed more affected by the phytoplankton biomass than by the experimental treatments. The highest biodiversity (1.40, by Shannon’s index) was found at salinity 6.

Dilution experiments in natural microbial communities are indeed a challenge, particularly in combination with acidification. We are aware that dilution with Milli-Q water does not mimic natural conditions and might have caused indirect treatment effects. Nevertheless, alternatives such as dilution with tap water or lake water were ruled out due to the addition of other compounds and different ions, with potential effects on the carbonate system, e.g. *A*_T_. Dilution of the ambient treatment followed by addition of salts was considered, but avoided because the addition of commercially available salts, different from the ions found in the Baltic Sea, had introduced yet another treatment factor. Despite the reduced buffering capacity (*A*_T_), we did not find any significant effects of *p*CO_2_ and we believe that it strengthens our arguments that the carbonate system was not that important in this study. Despite the addition of nutrients, the dilution resulted in lower nutrient concentrations in the lower salinity treatments. This makes it difficult to clearly separate a salinity effect from a nutrient effect. Nevertheless, the most pronounced increase in biomass was between Day 9 and Day 12 where elemental ratios (and nutrient concentrations) in all treatments showed a noticeable DIN and Si limitation. Our focus was diazotrophic cyanobacteria and the setup was advantageous for this group. However, also diatom biomass increased and was higher in the salinity 6 treatments on Day 12, in spite of DIN and Si limitation already on Day 9 (DIN:DIP ratio < 1.5; DIN:DIP:Si ratio < 1.9). Therefore, we argue that salinity did have an effect.

Both dinoflagellates and diatoms were negatively affected by lower salinity; thus, cyanobacteria like *Dolichospermum* spp. could get yet another competitive advantage in a future Baltic Proper. Our results suggest that the toxic *N. spumigena* will not grow as well in a lower salinity environment but, on the other hand, the increasing biovolume by Day 12 indicates acclimation. In laboratory studies, optimum salinity for growth of *N. spumigena* ranges from 7 (Mazur-Marzec et al. 2005) to 10 (Lehtimäki et al. 1997), but the species also grow well at both salinities 4 and 7 (Karlberg and Wulff 2013). For *Aphanizomenon* sp., Lehtimäki et al. (1997) concluded that *Aphanizomenon* sp. preferred salinity 0–5 over salinity 10 and 30, and when comparing salinity 7 and 4, no effects on biovolumes of *Aphanizomenon* sp. were detected (Karlberg and Wulff 2013). Elevated *p*CO_2_ had a positive effect on total biovolume for *Dolichospermum* spp. with the highest values found in the treatment with salinity 3 and elevated *p*CO_2_. For the toxic *N. spumigena*, lower biovolumes were found in salinity 3 compared to salinity 6, implying a less toxic future Baltic Sea. Moreover, the amount of fixed N_2_ and released NH_4_^+^ might increase, since *Dolichospermum* spp. showed relatively higher N_2_-fixation rates compared to *N. spumigena* (Klawonn et al. 2016), with a positive feedback mechanism on the microbial loop. Effects on copepods were revealed in a summer field survey where *N. spumigena* had positive effects on copepod egg production and egg viability, while *Aphanizomenon* sp. showed a negative relationship with egg viability (Hogfors et al. 2014. Thus, through lower biovolumes of *N. spumigena*, a future a less saline Baltic Proper could imply a less positive environment for copepods. In our study, no effect of the elevated *p*CO_2_ was observed for *N. spumigena* and *Aphanizomenon* sp., contradictory to Eichner et al. (2014b), showing a decreased growth rate in elevated *p*CO_2_ for *N. spumigena* and an increased growth rate at elevated *p*CO_2_ by Wannicke et al. (2012). Moreover, Brutemark et al. (2015) reported that no effects on growth of *Dolichospermum* spp. were found when exposed to low pH/high CO_2_. However, the latter three studies were performed on single-species cultures and, as shown by Mohlin et al. (2012), under stressful conditions growth rate of *N. spumigena* was stimulated by the presence of *Aphanizomenon* sp., further complicating interpretations from single-species experiments. Micrograzers were not affected by *p*CO_2_, which is consistent with results by Aberle et al. (2013) from a coastal planktonic community. *N. spumigena* has been assumed to be the only species of the three dominating filamentous cyanobacteria species in the Baltic Proper that produces toxin, but also *Dolichospermum* spp. has been proposed as a potential toxin producer in different parts of the Baltic Sea (Sivonen et al. 2007). The nodularin concentration has earlier shown to be affected by salinity (Lehtimäki et al. 1997; Mazur-Marzec et al. 2005). We measured nodularin on Days 0 and 12, but concentrations were always below or very close to the detection limit of the instrument. It is therefore unknown whether the treatments had any effects on the toxin concentration.

The specific treatment effects on heterotrophic bacteria (higher biomass in present day conditions) are difficult to distinguish in experiments with natural communities where bacteria cannot be tested separately from autotrophs. In our setup, the bacteria sampled were associated with the phytoplankton. The abundance of heterotrophic bacteria was, for example, positively correlated to *N. spumigena* and a negative treatment effect on this species would, thus, negatively affect the associated bacteria. Furthermore, environmental conditions and treatments stressing the phytoplankton community could result in more dissolved organic carbon (DOC) available for the heterotrophic bacteria, leading to increased abundance (like we observed between Days 0 and 3). This was observed in a large ocean acidification study where the heterotrophic activity was closely coupled to the primary productivity and release of DOC (Engel et al. 2013). In another mesocosm study, Grossart et al. (2006) report the indirect effects of *p*CO_2_ on heterotrophic bacteria mediated by the phytoplankton community. On the other hand, Endres et al. (2014) found a stimulation of bacterial growth at elevated *p*CO_2_ (lower pH). This stimulation was attributed to increased availability of gel particles as food source and substrate, plus enhanced enzymatic hydrolysis of organic matter. In the Baltic, however, Lindh et al. (2013) found that ocean acidification (lowering of 0.4 pH units) did not affect the biomass of a heterotrophic bacterial assemblage, neither alone or in combination with increased temperature, but ocean acidification in combination with increased temperature resulted in a shift in the bacterial community composition. These results highlight the complexity of unravelling the effects of climate change on natural microbial communities and further stress the importance of a multifactorial experimental approach. Moreover, salinity has been shown to affect both functional performance and composition of bacterial communities regardless of DOC composition (Langenheder et al. 2003). The heterotrophic bacterial assemblage in the Baltic Proper is typically an assemblage adapted to this brackish environment with a pronounced influence of freshwater groups and lack of typical marine species (Riemann et al. 2008; Andersson et al. 2010; Herlemann et al. 2011). A future less saline Baltic Proper could increase the proportion of freshwater groups with yet unknown consequences for the marine food web (e.g. Herlemann et al. 2011); however, Nydahl et al. (2013) suggest that in a future warmer and wetter climate, the heterotrophic bacterial activity will increase, with increased coastal hypoxia as a possible outcome. The DOC pool in our experiment was initially diluted in the low-salinity treatments, potentially reducing substrate access for heterotrophic bacteria. Indeed, bacterial productivity appeared higher initially in the high-salinity treatments, but no significant differences remained after 12 days between the high- and low-salinity treatments, indicating that other factors than carbon availability limited bacterial growth.

The highest biodiversity (by Shannon’s index) was found at salinity 6, implying that a future Baltic Proper may host a lower phytoplankton biodiversity. Our result is a snapshot in time, but considering the biodiversity gradient in the Baltic Sea, following the salinity gradient with higher biodiversity in the south, which is worth emphasizing. Lower biodiversity generally implies a decreased resilience towards environmental (including anthropogenic) stress; however, if all species within a functional group respond similar to pressure, a higher biodiversity will not offer resilience (Hughes et al. 2005). For the type of microbial communities studied, the close coupling between phytoplankton and heterotrophic bacteria further complicates interpretation of experimental treatment effects. For heterotrophic bacteria, the experimental treatments (salinity and *p*CO_2_) from an ecological perspective could be considered as press disturbance, where the shift in phytoplankton composition and biomass was comparable to pulse disturbance (Shade et al. 2012) with different implications for the heterotrophic bacterial community resistance and/or resilience (Baho et al. 2012; Shade et al. 2012).

In our study, *A*_T_ increased slightly during the experiment. Addition of CO_2_ does not affect *A*_T_, but the exudation of organic substances containing basic functional groups could explain the observed pattern (cf Kim and Lee 2009). Thus, to better describe the carbonate system, other parameters such as dissolved inorganic carbon might be preferred in similar experiments (Gattuso et al. 2010; Schulz and Riebesell 2013). To reach target salinities, seawater of higher salinity was mixed with Milli-Q water, thus reducing the buffering capacity of the experimental water. Consequently, the *A*_T_ of the experimental water differed from that of Baltic seawater of similar salinities. The reduced buffering capacity could lower pH, but was not expected to affect the microorganisms in our experiment (cf Ploug 2008; Karlberg and Wulff 2013), and the pH change in situ over 24 h was between 7.79 and 8.42. In a yet to be submitted study on another Baltic microbial community (Karlberg et al. unpublished), *A*_T_ was reduced from approximately 1500–1000 µmol kg^−1^ SW, with no effect on the microorganisms. In the aquaria, the diurnal variability in photosynthesis (i.e. carbon uptake) resulted in large variations in *p*CO_2_ levels, despite the constant supply with CO_2_-enriched air. Our measurements were performed in the morning and provide a snapshot of the carbonate system. Large variations of *p*CO_2_ due to the diurnal cycle of primary productivity have also been observed in coastal surface waters (Borges and Frankignoulle 1999; Fransson et al. 2004; Schulz and Riebesell 2013). Despite the complexity, maintaining a constant *p*CO_2_ in the medium lacks ecological relevance when performing CO_2_ enrichment experiments on primary producers. Our experimental setup generates a diurnal variable *p*CO_2_, fluctuating with similar wavelengths as in situ conditions. Therefore, bubbling with CO_2_-enriched air is an advantageous method when studying organisms capable of substantial CO_2_ perturbation (Gattuso et al. 2010; Karlberg and Wulff 2013; Torstensson et al. 2013, 2015). Also, due to the high CO_2_ uptake, the *p*CO_2_ differed from the targeted level of 960 µatm (projected for future atmospheric *p*CO_2_). Hence, a more intense bubbling to keep a *p*CO_2_ of 960 µatm had stimulated an unrealistically high CO_2_ sink in the system. Forcing very acidic conditions to a system with high primary productivity will overestimate the effects of ocean acidification, as these blooming surface communities will never experience those high levels during the climate scenario that we simulated (due to intense primary productivity). In our opinion, it is more realistic to start with high CO_2_ levels before the bloom and let it decrease as the bloom develops, just as in natural systems. The headspace will still represent simulated atmospheric levels of ~ 960 µatm. Furthermore, we performed a pilot study to choose an appropriate flow rate and the higher flow rate was chosen to somewhat compensate for the increased primary productivity over time. Again, to illustrate the diurnal changes in the treatment aquaria and in situ, we performed ca. 30 h measurements where samples were taken and analysed every second hour.

Similar to our study, in a post-bloom Baltic microplankton assemblage, no CO_2_-related effects in neither inorganic nor organic N pool sizes, or particulate matter N:P stoichiometry were found (Paul et al. 2016). Although nutrient levels were low by Day 12, in situ nutrient concentrations (DIN, DIP) in the surface water at the sampling site were lower with typical values of 0.02–0.06 µM (DIN) and 0.02 µM (DIP). Our Si concentrations by Day 12, however, were generally lower compared to in situ values of 6–8.6 µM. Apart from our experimental treatments, the Baltic Proper is under pressure with increased internal loading of phosphorus, lowering DIN:DIP ratios which presumably benefit diazotrophic cyanobacteria (Wasmund 1997; Vahtera et al. 2007a). In our study, nutrients without nitrogen and silicate were added to further mimic summer conditions in the Baltic Proper without introducing DIP limitation, and DIN:DIP ratios at Days 3 to 12 (< 5) confirmed nitrogen limitation in all aquaria. However, also DIP decreased, which was most likely caused by the P-storing abilities of the cyanobacteria (Vahtera et al. 2007b; Mohlin and Wulff 2009; Olofsson et al. 2016). Again, despite the Si limitation indicated by the DIN:DIP:Si ratios, both cell numbers and biovolume of diatoms increased by the end of the experiment. It is also worth noticing that the highest chl *a* concentrations by Day 12 was found in the treatment with salinity 3 and *p*CO_2_ 960 µatm, that is, a treatment with the lowest initial nutrient concentrations.

Effects of increased temperature were not addressed in this study, but is an additional potential stress factor for the Baltic microbial community. For cyanobacteria, it is proposed that an elevated temperature will give cyanobacteria a competitive advantage over other phytoplankton groups (Paerl and Huisman 2008). Baltic filamentous cyanobacteria have been shown to benefit from elevated temperatures both in laboratory studies (e.g. Karlberg and Wulff 2013) and in models (Hense et al. 2013).

Conclusively, elevated *p*CO_2_ had no significant effects on the natural microplanktonic community except for higher biovolume of *Dolichospermum* spp. and lower biomass of heterotrophic bacteria. At the end of experimental period, heterotrophic bacteria were correlated to *N. spumigena.* Consistent with our findings, results from the large mesocosm experiment in the Gulf of Finland 2012 (Hornick et al. 2017; Lischka et al. 2017) highlights the complexity of studying plankton community responses to increased *p*CO_2_ levels. Considering the Baltic Proper, we do not expect any dramatic effects of increased *p*CO_2_ in combination with decreased salinity on the microplanktonic food web, but effects on additional size classes and trophic levels were not a part of this study. Lower salinity significantly affected cyanobacteria together with biovolumes of dinoflagellates, diatoms, ciliates and heterotrophic bacteria, with higher biovolume of *Dolichospermum* spp. and lower biovolume of *N. spumigena*, dinoflagellates, diatoms, ciliates and heterotrophic bacteria in reduced salinity. Although the salinity effects on diatoms were apparent, they could not clearly be separated from the influence of inorganic nutrients. In addition, we found a clear diurnal cycle in Δ*F*/*F*_m_′ and pH, but without significant treatment effects and also in situ we observed the same diurnal pattern (*p*CO_2_, pH). Our study lasted 12 days, allowing for several generations of the organisms studied, but can still be considered a short time study. For example, in a laboratory study over 7 months, Torstensson et al. (2015) concluded that long-term acclimation was crucial for the diatom studied. However, *any* experimental design implicates choices with potentially associated biases. To conclude with a remark from Riebesell and Gattuso (2015) with respect to ocean acidification research “The paramount challenge for our research community will therefore be to assimilate the growing knowledge in each of these diverging research branches into an integrated assessment of short- to long-term responses to multiple drivers and their underlying mechanisms at the level of organisms, populations, communities and ecosystems.” Thus, we believe that our study can add one piece to the complicated puzzle to reveal the combined effects of increased *p*CO_2_ and reduced salinity levels on the Baltic microplanktonic community.
